# Self-reported needs of people living with psychotic disorders: Results from the Australian national psychosis survey

**DOI:** 10.3389/fpsyt.2022.1013919

**Published:** 2022-09-09

**Authors:** Christine Migliorini, Ellie Fossey, Carol Harvey

**Affiliations:** ^1^Department of Psychiatry, Psychosocial Research Centre, The University of Melbourne, Melbourne, VIC, Australia; ^2^NorthWest Area Mental Health Service, NorthWestern Mental Health, Coburg, VIC, Australia; ^3^Department of Occupational Therapy, Monash University, Frankston, VIC, Australia

**Keywords:** needs assessment, psychotic disorders, health systems-community, planning-community health, outcome and process assessment, patient preference, delivery of health care

## Abstract

Person-centered care is a collaborative approach to health care. To provide effective, person-centered care to people living with severe mental illness, it is necessary to understand how people view their own needs. The Perceived Need for Care Questionnaire (PNCQ) was used in the Australian National Survey of High Impact Psychosis (SHIP) to deepen understanding and evaluate, at a population level, the needs of Australian adults living with psychotic illness. SHIP participants were 1,825 adults, aged 18–65 years, living with psychotic illness and in contact with public specialized mental health services across Australia in 2010. The survey package included demographic and clinical items, and various scales including the PNCQ appraising a comprehensive range of life domains. Logistic regressions measured the impact that various demographic, clinical and psychosocial independent variables (e.g., loneliness, health-related quality of life, disability, accommodation type) had on the likelihood of inadequately met PNCQ domain-related need. Over two-thirds of people living with psychosis reported at least two areas of unmet need for care despite most being in contact with mental health services. Work or using one's time and socializing, counseling, and self-care domains had the largest proportion of inadequately met needs (range between 49 and 57%). Feelings of loneliness and/or social isolation were significantly associated with unmet needs across all PNCQ domains, except for financial needs. Health-related quality of life was significantly associated with unmet needs across all domains, except for housing needs. Disability was significantly associated with unmet social, occupation (work or time use), housing and medication-related needs. Consumers view their needs for care as unmet across many life areas despite being in contact with mental health services. Loneliness, unmet psychosocial needs, and health-related quality of life appear strongly interconnected and warrant greater attention in the delivery of person-centered care for people living with psychosis. Support to address social, work or time use and housing related needs among people living with psychosis appears less well targeted toward those with disability. Results underscore the link between quality of life, recovery and needs. These inter-relationships should be considered in mental health services research and evaluation.

## Introduction

The World Health Organization (1) advocates person-centered care as “an approach to care that consciously adopts the perspectives of individuals, families and communities, and sees them as participants as well as beneficiaries of trusted health systems that respond to their needs and preferences in humane and holistic ways” (1, People-centered health services, para. 1). Person-centered care is otherwise known as, *Patient-centered care; Client-centered care; Consumer-directed care; Self-directed care* (2, 3) and regarded as integral to health care provision in Australia (4). Person-centered care emphasizes partnerships in which the health professionals and consumers are active partners in care, recognizing that individuals bring a range of skills and abilities, needs, and wants (5). To provide effective person-centered care, it is necessary to understand how people view their own needs (6).

In mental health care, person-centered care is advocated nationally and internationally in policy and service frameworks focused on recovery-oriented practice [e.g., (6, 7)]. In this context, a person-centered and recovery-oriented approach must recognize the complexity of health issues and other circumstances that people living with severe mental illness, including psychosis, face. People diagnosed with psychotic conditions frequently have comorbid mental and physical health conditions, as well as experiencing social difficulties and challenges (8–11). Therefore, discussions between mental health consumers and clinicians need to address both the mental health issue and the broader context to practice person-centered care (12).

Assessing the needs of individuals with mental illness has been of interest for many years. The Camberwell Assessment of Need questionnaire [CAN: (13)] was specifically developed to provide a comprehensive assessment of needs within 22 life domains and has been used in many studies with consumers experiencing severe mental illness [e.g., (14–16)]. Originally designed for clinical use, the questionnaire can be used as a structured exploration to identify problematic life domains for an individual or in service-related research, with available response choices of 0 = no serious problem; 1 = no serious problem or moderate problem because of continuing intervention (met need); and 2 = current serious problem (unmet need). Previous studies investigating how people view their needs for mental health care at a population level have more commonly been epidemiological surveys of common mental disorders with brief enquiries into treatment needs and/or center on service providers' perspectives of needs (17–19). Briefer than the CAN, the Perceived Need for Care Questionnaire (PNCQ) was specifically designed for epidemiological and health services research (20, 21) and has been used for surveys of high prevalence disorders, such as depression and anxiety. Until now, the PNCQ has not been used for epidemiological surveys of low prevalence disorders. Like the CAN, the PNCQ produces categories of no need, unmet need and met needs but adds the category of partially met need, though it does not rate the seriousness of the need as the CAN does.

Several Australian studies have sought to understand the views of adults regarding their needs for care, some of whom were living with psychosis. For example, this includes a study that explored mental health-related needs as perceived by attendees in primary and mental health care settings, being a general practice clinic and area mental health service (AMHS), respectively (22). This mixed methods study incorporated the PNCQ and semi-structured interviews to explore the meanings behind the self-identified needs of participants, a little over half of whom reported a diagnosis of a mental illness (e.g., depression, schizophrenia, bipolar disorder). Not unexpectedly, compared to general practice participants, AMHS participants (*n* = 23) reported the larger range of needs: most participants nominated mental health-related needs covering medication, mental health-related information and counseling, social interventions addressing housing and finances, and skills development-related needs concerning looking after oneself, one's home and use of time. While these results are of interest, the modest sample does limit confidence in their generalizability. A more recent example is an Australian study that investigated the needs of consumers and carers from diverse backgrounds in a family psychoeducation intervention for people experiencing severe mental illness (including major depression, bipolar disorder, and schizophrenia) and their families (23). Qualitative analysis of responses to Carers' and Users' Expectations of Services questionnaires from this study found similar needs comprising illness related information (illness course, treatment, service options), relationship needs (improving communications and reducing conflicts within their families) and social integration-related needs (addressing finances, lack of time and social barriers to social engagement) (23).

A substantial gap in current knowledge of Australian service provision exists at a population level in relation to the perceived needs for care of those living with psychosis. A large population-based survey of Australian adults living with psychotic illness provided the opportunity to gain insight into their identified needs and how well those needs were met over a 12-month period. This paper will explore three broad areas from within the survey, using the following six research questions:

### Perceived needs distribution

1. What were the perceived needs reported by persons living with psychosis?
2. Were there any links between the various types of need for care?

### Meeting needs for care

3. Over the previous year, who were perceived to have spent the most time providing help for their mental health problems?
4. Over the previous year, who were perceived to have been the most helpful for their mental health?
5. How well were mental health services perceived to meet their identified needs?

### Predictors of the need for care

6. Were there psychosocial factors associated with the likelihood of a need being rated as partially or wholly unmet i.e., what factors, if any, were associated with the existence of inadequately met need?

## Materials and methods

The Australian National Survey of High Impact Psychosis (SHIP) was a large population-based study of persons living with psychotic illness who were in contact with public specialized mental health services, clinical or NGO, recruited in seven catchment sites across Australia. All participants (*N* = 1,825) were adults aged 18 to 65 years. The survey package contained 32 modules, covering socio-economic status characteristics and other personal and clinical details as well as various scales, including the PNCQ. Detailed descriptions of the recruitment sites, methodology, inclusion and exclusion criteria and the full survey package can be found elsewhere [e.g., (11)]. The variables used in this current study include demographic, personal and clinical characteristics of the individuals participating in SHIP, and published scales, all of which were collected and completed during an interview with a trained clinical researcher as part of the SHIP study (11). The scales are described below.

### Perceived need for care questionnaire

The Perceived Need for Care Questionnaire (PNCQ) (20) canvasses a person's own view concerning their needs for mental health and other services. Eight domains are appraised for met, partially met (received some help but did no receive as much help as they perceived they needed) and unmet need (would have liked help but did not receive any help) as well as no need (did not want any help) over the previous 12-months. As integrated into the SHIP survey package, the eight PNCQ domains included: (1) Social needs which refers to help to socialize; (2) Work/Time Use needs which refers to help to work or use time; (3) Financial needs which refers to financial help; (4) Housing needs which refers to help to sort out housing; (5) Self-care needs which refers to help to look after self or home; (6) Mental Health (MH) Information needs which refers to the receipt of any information about mental illness, treatment and services available; (7) Medication needs which refers to the receipt of medicine or tablets for mental health; and (8) Counseling needs which refers to the receipt of any counseling or other talking therapy such as psychotherapy or group therapy. For the bivariate analyses examining associations between need types and for the multivariate logistic analyses, the dependent variable of Current Need was created whereby unmet and partially met needs categories were collapsed into inadequately met need = 1, and needs met, and no needs were collapsed into adequately met need = 0.

### Multidimensional scale of independent functioning

The Mulitdimensional Scale of Independent Functioning (MSIF) (24) is a scale developed for use with psychiatric outpatients assessing the relative level of disability experienced, focusing on role performance and activities of daily living. The MSIF consists of three subscales and an overall global independent functioning outcome rating which reflects the extent of disability considering the level of support used within the environments of work, education, and residence. The scale is partitioned into 1 = essentially normally functioning, 2 = very mild disability, 3 = somewhat disabled, 4 = moderately disabled, 5 = significantly disabled, 6 = extremely disabled, and 7 = totally disabled. For this study, the range was collapsed into No disability (was score 1), Mild disability (was scores 2 and 3), Moderate disability (was score 4), and Severe disability (was scores 5 to 7) for descriptive purposes, and None/mild disability (was scores 1–3) and Moderate/Severe disability (was scores 4–7) for the multivariate analyses.

### Assessment of quality of life

The Assessment of Quality of Life (AQoL) is a valid and sensitive health-related quality of life (HRQoL) instrument (25). It comprises five primary dimensions being illness, independent living, physical ability, psychological wellbeing, and social relationships. Raw scores can be summed to produce subcategory or dimensional scores (not used here) as well as a total score. Total scores can range from 0 to 45 where “0” represents good HRQoL and “45” the worst possible HRQoL score.

### Additional composite variables

Five additional composite variables from the SHIP survey were used in this study: Support providers-Most help provided, Most time provided, Comorbidity, Loneliness and Accommodation, as described below.

#### Support providers: Most time provided and most help provided

The following items were asked of participants (a) In the last 12 months who in your opinion spent the most time providing you with help for your mental health problems? And (b), In the last 12 months who in your opinion was the most helpful? The response choices for both items were GP, psychiatrist (public or private), psychologist, MH nurse, case manager, other mental health professional, family/friend, complementary or alternative therapist, other and no one. The response choices of psychiatrist (public or private), psychologist, MH nurse, case manager and other mental health professional, were collapsed into the single subcategory of MH Professional. The response choices of complementary or alternative therapist (time = 0.2%, help = 0.2%) and an undefined other (time = 2.5%, help = 5.3%) were deemed too infrequently endorsed and dissimilar in content to combine and therefore excluded from the study.

#### Comorbidity

A yes response (scored as 1) to any of the following range of physical conditions was summed to provide the composite independent variable - Comorbidity:

- Arthritis
- Asthma
- Epilepsy
- Stroke/Transient Ischemic Attack
- Heart attack
- Angina
- Other heart disease (e.g., arrhythmias)
- Hepatitis
- Other liver disease
- Kidney disease
- Anemia
- Memory problems
- Respiratory problems
- Head injury–lost consciousness
- Parkinson's disease
- Frequent or severe headaches/migraines
- Eating disorders such as anorexia or bulimia nervosa
- Chronic back neck or other pain
- Allergies
- Cancer
- Congenital disorders/syndromes

#### Loneliness

The following item was asked of participants: In the last 12 months, have you felt lonely? Response choices were (a) I have plenty of friends, and have not been lonely, (b) Although I have friends, I have felt lonely occasionally, (c) I have some friends but have been lonely for company, and (d) I have felt socially isolated and lonely. These response choices were abridged into (a) 0 = Not lonely, (b and c) 1 = Lonely, and (d) 2 = Socially Isolated & lonely.

#### Accommodation

The response choices for this independent variable were collapsed into three subcategories: (a) Own/rented comprising the subgroups of family home, private residence, rental private residence, rental public residence or living with friends; (b) Formally managed and shared accommodation comprising the subgroups of supported residential, institution, private boarding house/room etc. for the general population, private boarding house/room for people with a disability, 24 h supported community accommodation; and (c) Homeless or unstable accommodation comprising the subgroups of homeless/no fixed abode, caravan/houseboat, crisis short-term, hospital, prison, and other.

### Analyses

IBM SPSS v26 (IBM SPSS Statistics, RRID:SCR_016479) was used for the statistical analyses and figures were produced using MS Excel 2016 (Microsoft Excel, RRID:SCR_016137). Summary descriptive statistics (counts, percentages, means, standard deviations and range) were calculated. When exploring the broad area of distribution of needs for care, participants reporting no need were excluded from those analyses. A series of non-parametric bivariate analyses (Chi Square with Yates Continuity Correction: X^2^) were conducted to explore relationships between the various need types. Strength of the relationships (phi coefficient effect size: ϕ) was based on Cohen's criteria of 0.10 to 0.29 for small effect, 0.30 to 0.49 for medium effect and 0.50 to 1.0 for large effect (26).

Non-parametric analysis (Chi Square Test for Independence) was used to explore the relationship between supporters rated as helping the most and those who spent the most time helping. Given the focus, participants were excluded from the analysis if they expressed no need linked to each PNCQ domain. Since there were four subcategories in both variables, Cramer's V was used to indicate the size of the effect; the recommended interpretation of Cramer's V with four categories used is small = 0.06, medium = 0.17 and large = 0.29 (26). The minimum expected cell frequency assumption was not violated in any Chi Square Test.

Direct binomial logistic regressions were performed to assess the impact of various demographic, clinical and psychosocial independent variables on the likelihood that participants would report a need being inadequately met (partially met and unmet) need per PNCQ domain. Given the focus was on factors linked with inadequately met need compared to others, the full sample was used in these analyses. No outliers (SD ≥ 2.5) were found in any models.

*P*-values < 0.05 were deemed statistically significant across all statistical analyses. Ethics approval for the project was received from all relevant University and Health Network Human Ethics Committees, and the study was conducted in accordance with the Declaration of Helsinki.

## Results

### The participants

Sixty percent were male, as shown in Table 1. Participants' mean age was 38 years and ranged from 18 to 65 years. Most were single, living in their own or rental accommodation and experiencing mild or moderate levels of disability in independent functioning. Their average length of psychotic illness was 15 years but ranged from less than one to over 50 years. Most participants had received a diagnosis of either schizophrenia or schizoaffective disorder. Diagnoses are reported for descriptive purposes only, and not for further analyses (see Table 1).

**Table 1:** Descriptive characteristics of the participant sample (*N* = 1,825).

**Independent variable**	**Categories**	**Count (%)**
**Sex**		
	Male	1,087 (59.6%)
	Female	738 (40.4%)
**Marital status**		
	Single	1,117 (61.2%)
	Married/defacto	312 (17.1%)
	Divorced/widowed	396 (21.7%)
**Current accommodation type**		
	Own/rented	1,492 (81.8%)
	Formally managed accommodation	265 (14.5%)
	Homeless or unstable accommodation	68 (3.7%)
**Comorbidity**	No comorbid conditions	307 (16.9%)
	1-2 comorbid conditions	696 (38.1%)
	3-15 comorbid conditions	813 (44.8%)
**Disability (MSIF)**		
	No disability	105 (5.8%)
	Mild disability	803 (44.0%)
	Moderate disability	502 (27.5%)
	Severe disability	415 (22.7%)
**Loneliness**		
	Not lonely	354 (19.9%)
	Lonely	1,017 (55.7%)
	Socially isolated & lonely	409 (22.4%)
**ICD-10 diagnosis**		
	Schizophrenia	857 (47.0%)
	Schizoaffective disorder	293 (16.1%)
	Bipolar disorder	319 (17.5%)
	Depressive psychosis	81 (4.4%)
	Delusional and non-organic psychosis	92 (5.0%)
	Other	183 (10.1%)
	**Mean (SD)**	**Range**
Age at time of interview	38.4 years (11.2 years)	18–65 years
Age at onset of mental illness	23.7 years (8.6 years)	6–62 years
Duration of Psychotic illness	14.7 years (10.3 years)	<1–50 years
Overall AQoL	7.5 (4.7)	0–30

Some variable counts do not sum to 1,825 due to missing data; Screen positive for psychosis but did not meet full criteria for ICD-10 psychosis; MSIF, Multidimensional Scale of Independent Functioning; AQoL, Assessment of Quality of Life; SD, Standard Deviation.

### Perceived needs distribution

**Figure 1:**
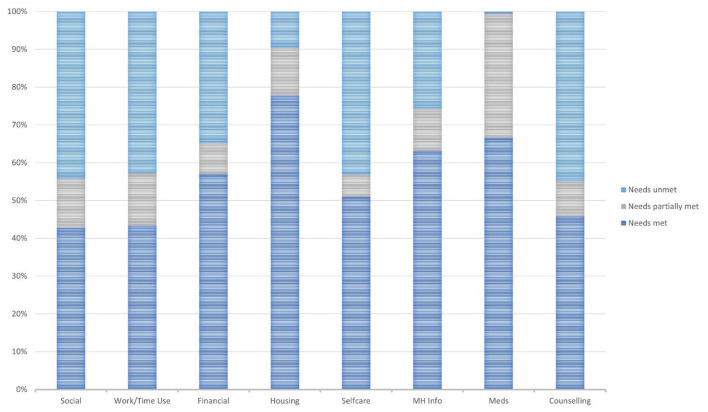
Percentage of participants who reported a need per PNCQ domain.

This section focuses on only those participants who expressed a need whether fully or partially met or not i.e., participants who expressed no need were excluded from consideration, per PNCQ domain. Figure 1 displays the relative responses within each of the PNCQ domains. Medication needs (67%), MH Information (63%) and Housing needs (78%) were the three domains where the highest proportion of perceived needs were met. There is a caveat to the interpretation of Housing needs i.e., only those who moved residence in the previous 12 months were asked the PNCQ Housing assistance-related items, otherwise a skip was built into the survey. Consequently, only 430 participants or 24% of the full sample were asked the housing assistance-related items. Many participants expressed a range of unmet or partially met needs in other PNCQ domains, with only 43% of Social needs met, 43% of Work/Time Use needs met, 51% of Self-care needs met and 46% of Counseling needs met (see Figure 1).

**Figure 2:**
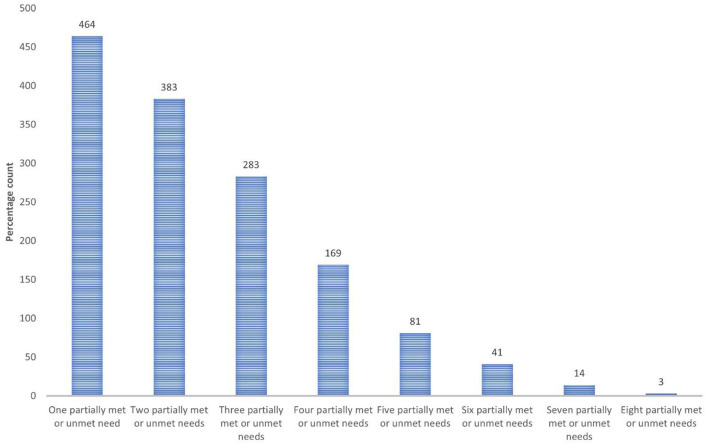
Frequency of partially met or unmet needs reported by participants who wanted assistance.

Among participants who expressed any need, most reported multiple needs i.e., 67.7% of participants expressed needs for care in two or more areas that were only partially met or unmet (see Figure 2). A series of Chi-square test for independence (with Yates Continuity Correction) analyses (χ^2^) were used to explore relationships between PNCQ domain needs - met and inadequately met (partially met and unmet). Several links were found between most need types meaning when one unmet need existed another unmet need type could co-occur; these associations were weak with effect sizes (Phi) often below 0.3 (the threshold for medium effect) (26). Medium sized positive connections were found between Social and Work/Time Use related needs, and between MH Information and Counseling needs. A further association between Housing and Financial needs approached medium effect size with Phi being 0.293 (see Table 2).

**Table 2:** Series of bivariate analyses (Chi Square with Yates Continuity Correction: X^2^) displaying the relationships between the PNCQ domains.

	**Social**	**Work / Time use**	**Financial**	**Housing**	**Self-care**	**MH Info**	**Meds**	**Counseling**
Social	1	χ^2^ (1, *n* = 577) = 53.46, *p* < 0.0005 ϕ = 0.308	χ^2^ (1, *n* = 532) = 11.71, *p* < 0.001 ϕ = 0.152	χ^2^ (1, *n* = 241) =8.31, *p = 0.0*04 ϕ = 0.196	χ^2^ (1, *n* = 525) = 39.44, *p* < 0.0005 ϕ = 0.278	χ^2^ (1, *n* = 762) = 22.87, *p* < 0.0005 ϕ = 0.176	χ^2^ (1, *n* = 922) = 6.41, *p* = 0.011 ϕ = 0.086	χ^2^ (1, *n* = 593) = 22.83, *p* < 0.0005 ϕ = 0.200
Work/Time Use		1	χ^2^ (1, *n* = 543) = 15.80, *p* < 0.0005 ϕ = 0.174	χ^2^ (1, *n* = 262) = 5.31, *p* = 0.021 ϕ = 0.152	χ^2^ (1, *n* = 495) = 30.64, *p* < 0.0005 ϕ = 0.253	χ^2^ (1, *n* = 712) = 27.93, *p* < 0.0005 ϕ = 0.201	χ^2^ (1, *n* = 866) = 8.44, *p* = 0.004 ϕ = 0.101	χ^2^ (1, *n* = 544) = 24.72, *p* < 0.0005 ϕ = 0.217
Financial			1	χ^2^ (1, *n* = 248) = 19.96 *p* < 0.001 ϕ = 0.293	χ^2^ (1, *n* = 473) = 33.23, *p < * 0.001 ϕ = 0.269	χ^2^ (1, *n* = 692) = 22.65, *p* < 0.001 ϕ = 0.184	χ^2^ (1, *n* = 842) = 13.74, *p* < 0.001 ϕ = 0.130	χ^2^ (1, *n* = 522) = 14.46, *p* < 0.001 ϕ = 0.170
Housing				1	χ^2^ (1, *n* = 204) = 8.67, *p* = 0.003 ϕ =0.219	χ^2^ (1, *n* = 340) = 5.75, *p* = 0.016 ϕ = 0.138	χ^2^ (1, *n* = 407) = 2.36, *p* = 0.124 ϕ = 0.082	χ^2^ (1, *n* = 230) = 0.000, *p* = 1.00 ϕ = −0.006
Self-care					1	χ^2^ (1, *n* = 628) = 19.48, *p* < 0.0005 ϕ = 0.179	χ^2^ (1, *n* = 743) = 1.92, *p* = 0.166 ϕ = 0.054	χ^2^ (1, *n* = 471) = 36.60, *p* < 0.0005 ϕ = 0.283
MH Info						1	χ^2^ (1, *n* = 1,296) = 30.81, *p* < 0.0005 ϕ = 0.156	χ^2^ (1, *n* = 823) = 99.77, *p* < 0.001 ϕ = 0.351
Meds							1	χ^2^ (1, *n* = 934) = 14.13, *p* < 0.0005 ϕ = 0.125
Counseling								1

Effect size phi coefficient: ϕ; Cohen's criteria for effect size: 0.10 to 0.29 for small effect, 0.30 to 0.49 for medium effect and 0.50 to 1.0 for large effect.

### Meeting needs for care

Overall, a significant association was found between those who provided the most help for mental health problems over the previous 12 months and those who provided the most time, χ^2^ (9, *n* = 1,312) = 2,341.05, *p* < 0.0005, Cramer's V = 0.771 which was a large effect size (26). Those people identified as spending the most time providing help were also generally viewed as the most helpful. Thus, mental health professionals who spent the most time providing help were perceived as the most helpful by 91% of those participants; GPs spending the most time providing help were seen as most helpful by 77% of those participants; and when family and friends spent the most time providing help, this was endorsed as most helpful by 81% of those participants (see Table 3).

**Table 3:** Relationship between supporter time and supporter helpfulness as perceived by participants.

	**Most helpful**					
**Most time provided**		**No-one**	**GP**	**MH Prof**	**Family/Friend**	**Total**
	No-one	84	4	8	13	109
	GP	1	72	18	6	97
	MH Prof	10	15	682	51	758
	Family/Friend	4	3	38	303	348
	Total	99	94	756	373	1,312

### Predictors of the need for care

Given the dominance of weak links between several need for care types, direct binary logistic regression analyses were undertaken to ascertain what factors might help predict whether a need for care might be inadequately met (i.e., partially, or wholly unmet needs) in contrast to when a need was either met or no need was reported. For this series of analyses, the dependent variable of interest was inadequately met need per PNCQ domain, and the full sample was used. Each model contained ten independent variables (Sex, Marital Status, Age, Duration of psychotic illness, Comorbidity, Accommodation type, overall AQoL, Disability, Loneliness, Most Help Provided) (see Table 4). The full model containing all predictors was statistically significant for all eight PNCQ domain regressions (see Table 5); however, the factors that made statistically significant contributions differ for each need for care domain. See Table 4 and as described below.

**Table 4:** Binary logistic regressions (all sample): Odd ratios (OR) and confidence intervals (CIs) predicting the outcomes of *inadequately or unmet need* response per PNCQ domain.

**IV predictors**		**Social** **(*n* = 1,580)** ***OR*** **(95% *CI*)**	**Work/Time use (*n* = 1,563) *OR* (95% *CI*)**	**Financial** **(*n* = 1,585)** ***OR*** **(95% *CI*)**	**Housing (*n* = 434) OR (95% CI)**	**Self-care** **(*n* = 1,576)** ***OR*** **(95% *CI*)**	**MH Info (*n* = 1,563) *OR* (95% *CI*)**	**Meds** **(*n* = 1,588)** **OR** **(95% CI)**	**Counseling (*n* = 1,531) *OR* (95% *CI*)**
Sex	Male	*Ref*	*Ref*	*Ref*	*Ref*	*Ref*	*Ref*	*Ref*	*Ref*
	Female	1.04 (0.82–1.32)	1.00 (0.79–1.28)	0.93 (0.71–1.22)	0.87 (0.50–1.50)	0.95 (0.72–1.24)	1.14 (0.89–1.46)	**1.29 (1.02–1.64)**	**1.35 (1.06–1.73)**
Marital status	Married/defacto	*Ref*	*Ref*	*Ref*	*Ref*	*Ref*	*Ref*	*Ref*	*Ref*
	Single	1.01 (0.73–1.40)	0.91 (0.66–1.26)	**0.66 (0.47–0.93)**	0.50 (0.24–1.08)	0.83 (0.58–1.19)	0.99 (0.71–1.37)	0.78 (0.58–1.07)	0.97 (0.71–1.34)
	Divorced/widowed	1.10 (0.77–1.58)	1.10 (0.76–1.59)	0.96 (0.65–1.41)	0.68 (0.28–1.63)	1.08 (0.72–1.60)	0.84 (0.58–1.22)	0.93 (0.65–1.32)	0.87 (0.61–1.25)
Current age (years)		0.99 (0.98–1.01)	**0.97 (0.96–0.99)**	**0.98 (0.96–0.99)**	1.00 (0.96–1.04)	**0.98 (0.96–0.99)**	1.01 (1.00–1.03)	0.98 (0.97–1.00)	1.00 (0.98–1.01)
Duration of psychotic illness (years)		1.01 (0.99–1.02)	1.00 (0.98–1.02)	1.00 (0.98–1.01)	0.98 (0.94–1.01)	**1.02 (1.00–1.04)**	0.99 (0.97–1.00)	1.00 (0.98–1.01)	0.99 (0.98–1.01)
Physical comorbidity	No comorbid	*Ref*	*Ref*	*Ref*	*Ref*	*Ref*	*Ref*	*Ref*	*Ref*
	1-2 comorbid	1.00 (0.72–1.40)	0.80 (0.58–1.10)	1.26 (0.86–1.84)	1.35 (0.65–2.79)	1.19 (0.80–1.78)	0.80 (0.76–1.12)	1.24 (0.89–1.73)	1.03 (0.73–1.45)
	3–15 comorbid	0.88 (0.62–1.25)	0.76 (0.54–1.07)	1.16 (0.78–1.73)	1.69 (0.76–3.72)	1.09 (0.72–1.65)	1.06 (0.75–1.51)	1.27 (0.90–1.80)	1.23 (0.88–1.76)
Accommodation type	Own/family home	Ref	Ref	Ref	Ref	Ref	Ref	Ref	Ref
	Formally managed accommodation	0.82 (0.58–1.15)	0.84 (0.60–1.18)	1.00 (0.69–1.74)	0.62 (0.31–1.25)	**0.56 (0.37–0.85)**	1.13 (0.80–1.59)	1.06 (0.76–1.48)	0.88 (0.61–1.26)
	Homeless or unstable accommodation	1.70 (0.96–2.99)	0.99 (0.56–1.76)	1.74 (0.97–3.11)	1.80 (0.72–4.50)	0.77 (0.40–1.49)	0.91 (0.49–1.69)	0.84 (0.47–1.51)	1.12 (0.62–2.03)
AQoL		**1.07 (1.04–1.11)**	**1.06 (1.03–1.09)**	**1.07 (1.04–1.11)**	0.96 (0.90–1.03)	**1.11 (1.08–1.15)**	**1.03 (1.00–1.06)**	**1.06 (1.03–1.09)**	**1.06 (1.03–1.09)**
Loneliness	Not lonely	*Ref*	*Ref*	*Ref*	*Ref*	*Ref*	*Ref*	*Ref*	*Ref*
	Lonely	**2.16 (1.52–3.06)**	1.37 (0.99–1.89)	1.30 (0.90–1.87)	1.87 (0.81–4.30)	**1.52 (1.02–2.26)**	1.28 (0.92–1.71)	1.07 (0.79–1.45)	**1.73 (1.23–2.42)**
	Isolated	**3.21 (2.14–4.82)**	**1.64 (1.11–1.70)**	1.21 (0.79–1.87)	**3.59 (1.40–9.19)**	1.54 (0.98–2.43)	**1.68 (1.13–2.50)**	**1.54 (1.07–2.23)**	**1.80 (1.20–2.70)**
Disability	Norm/mild	*Ref*	*Ref*	*Ref*	*Ref*	*Ref*	*Ref*	*Ref*	*Ref*
	Moderate/severe	**1.39 (1.09–1.78)**	**1.33 (1.04–1.70)**	0.93 (0.70–1.22)	**2.25 (1.28–3.97)**	1.17 (0.89–1.43)	1.27 (0.30–0.74)	**1.29 (1.02–1.65)**	0.91 (0.71–1.17)
MH support -most help	No-one	*Ref*	*Ref*	*Ref*	*Ref*	*Ref*	*Ref*	*Ref*	*Ref*
	MH Prof	0.82 (0.51–1.30)	**0.61 (0.39–0.95)**	**0.56 (0.35–0.89)**	0.68 (0.23–2.04)	**0.51 (0.32–0.83)**	**0.47** **(0.30** **−0.74)**	0.65 (0.42–1.01)	**0.60 (1.19–0.94)**
	Family/Friend	1.14 (0.70–1.86)	0.80 (0.51–1.28)	0.68 (0.41–1.10)	0.65 (0.21–2.04)	0.74 (0.45–1.22)	0.95 (0.60–1.52)	0.96 (0.61–1.51)	1.19 (0.74–1.90)
	GP	0.95 (0.51–1.75)	0.70 (0.39–1.28)	0.54 (0.28–1.04)	0.45 (0.07–2.77)	0.80 (0.42–1.51)	0.80 (0.45–1.44)	0.64 (0.35–1.16)	1.12 (0.63–2.01)
Constant		0.15	1.02	0.57	0.27	0.26	0.28	0.52	0.25

AQoL, Assessment of Quality of Life; MH, Mental Health; GP, General Practitioner; IV, Independent variable; Statistically significant results in bold font.

**Table 5:** Logistic regression models statistics.

		**Social**	**Work/Time use**	**Financial**	**Housing**	**Self-care**	**MH Info**	**Meds**	**Counseling**
**Significance of model**	**χ^2^ ***p***-value**	140.56 *p* < 0.0005	81.33 *p* < 0.0005	64.36 *p* < 0.0005	27.54 *p* < 0.036	117.70 *p* < 0.0005	79.76 *p* < 0.0005	87.76 *p* < 0.0005	100.09 *p* < 0.0005
Model percentage variance predicted	Between Cox and Snell R^2^ and Nagelkerke R^2^	8.5–11.9%	5.0–7.2%	4.0–6.1%	6.1–9.8%	7.2–11.1%	4.6–6.6%	5.3–7.5%	6.3–8.9%
Percentage of cases correctly classified		69.30%	70.70%	78.40%	80.50%	75.50%	73.20%	68.10%	69.40%

χ^2^, Chi-square goodness of fit test; MH Info, Mental Health Information; Meds, Medication.

#### Social need

Three of the independent factors made a unique statistically significant contribution to the model (AQoL, Loneliness and Disability). The strongest predictor of reporting inadequately met social need was isolation, indicating that participants who reported their social needs being inadequately met were more than three times or 321% more likely to also perceive themselves as socially isolated, and a little more than twice as likely or 216% to describe feeling lonely, controlling for other factors. Each unit increase in AQoL score (i.e., poorer HRQoL) also increased the likelihood of inadequately met social need by 7%. Further, participants with moderate/severe disability were nearly 40% more likely to report their social needs to be inadequately met, controlling for other factors (see Table 4).

#### Work/time use need

Five independent factors made a unique statistically significant contribution to the model (Age, AQoL, Loneliness, Disability and Most Help Provided). The strongest predictors of reporting inadequately met work/time use related needs were feeling isolated and supporter providing most help. Participants who perceived themselves as socially isolated and lonely were 64% as likely to also report their work/time use needs being inadequately met, controlling for other factors. Conversely, inadequately met work/time use needs decreased by 64% when the supporter identified as providing the most help was a MH professional. Each unit increase in AQoL score also increased the likelihood of inadequately met work/time use need by 6%. Each yearly increase in age decreased the likelihood of work/time use related need being inadequately met by 3%. Moderate/severe disability increased the likelihood of work/time use need reported as inadequately met by 33%, controlling for other factors (see Table 4).

#### Financial need

Four independent factors made statistically significant contributions to the model (Marital status, Age, AQoL and Most Help Provided). Each yearly increase in age decreased the likelihood of financial need being inadequately met by 2%. Each unit increase in AQoL (whereby higher AQoL scores indicate poorer HRQoL) increased the likelihood of financial need being inadequately met by 7%. When mental health professionals were identified as being the most helpful, then the likelihood of financial needs being inadequately met reduced by around 79%, all other factors being equal. The likelihood of inadequately met financial need also significantly decreased by 50%, when participants were single, all other factors being equal (see Table 4).

#### Housing need

Two independent factors made statistically significant contributions to the model (Loneliness and Disability). Participants experiencing moderate/severe disability were 225% more likely to report their housing needs to be inadequately met. Being social isolated increased the likelihood of experiencing inadequately met housing needs by over three and a half times (359%), all other factors being equal (see Table 4).

#### Self-care need

Six independent factors made statistically significant contributions to the model, three increasing the likelihood (Duration of psychotic illness, AQoL, Loneliness), and three decreasing the likelihood (Age, Accommodation, Most Help Provided) of inadequately met self-care needs. Each year increase in illness duration increased the likelihood of self-care needs being inadequately met by 2%. For each unit increase in AQoL score and therefore poorer HRQoL, the likelihood that the participant self-care needs were also inadequately met increased by 11% and feeling lonely also increased the likelihood of inadequately met need by 52%, all other factors being equal (see Table 4).

Conversely, every year older in age decreased the likelihood that self-care needs were inadequately met by 2%. Residing in institutionally related accommodation decreased the likelihood of inadequately met need by 79% and when MH professionals were identified as most helpful then the likelihood of inadequately met self-care needs reduced nearly twofold (96%) (see Table 4).

#### MH information need

Three factors made a unique statistically significant contribution to the model (AQoL, Loneliness, Most Help Provided). Participants who reported being socially isolated were 68% more likely to report that their MH information needs were inadequately met compared to those who were not lonely, controlling for other factors. Each unit increase in AQoL score and therefore poorer HRQoL, increased by 3% the likelihood of MH information needs being inadequately met. When MH professionals were identified as providing the most help, then participants were more than twice as likely or 213% more likely to have had their MH information needs met, all other factors being equal (see Table 4).

#### Medication need

Four factors made a unique statistically significant contribution to the model (Sex, AQoL, Loneliness and Disability). Females were 29% more likely to report that their need for medication was inadequately met. Participants reporting moderate/severe disability were 29% more likely to report medication needs to be inadequately met. Each unit increase in AQoL score and therefore poorer HRQoL, increased the likelihood of medication need being inadequately met by 6%. Finally, being socially isolated increased the likelihood of inadequately met need for medication by 54%, all other factors being equal (see Table 4).

#### Counseling need

Four factors made a unique statistically significant contribution to the model (Sex, AQoL, Loneliness, and Most Help Provided). Females were 35% more likely to report their counseling needs to be inadequately met compared to males, all other factors being equal. Each unit increase in AQoL score and therefore poorer HRQoL, increased by 6% the likelihood of counseling needs being inadequately met. Feeling lonely and being socially isolated significantly increased the likelihood of counseling needs being inadequately met, by 73 and 80%, respectively. When MH professionals were identified as providing the most help, then participants were 67% more likely to have had their counseling needs met, all other factors being equal (see Table 4).

## Discussion

This is one of few studies internationally to systematically investigate diverse categories of perceived need in a large population-based sample of people living with psychotic disorders. Most participants reported at least two areas of inadequately met need. Perhaps unsurprisingly in a prevalence sample ascertained within treatment services (11), the lowest proportion of wholly unmet needs was in the medication domain and participants reported that mental health practitioners provided the most help and support for their mental health problems. Nonetheless, family and friends also made important contributions, as is increasingly acknowledged in research, policy, and practice [e.g., (27–30)]. This is consistent with a longitudinal study of Canadians with severe mental disorders, including psychoses, which suggested that changes in perceived adequacy of help mostly involved services use, as well as help received from relatives (29).

Few participants in the present study reported inadequately met needs concerning housing, which may reflect that these needs were only explored in relation to finding and moving house, for which assistance is typically available, whereas unmet housing needs more broadly defined can also reflect needs related to looking after one's home (16). In the current study except for housing needs, HRQoL appears to show important associations with unmet need. Conclusions about the directionality of this effect cannot be derived from this study, but greater unmet needs have been associated with poorer quality of life, in previous cross-sectional and longitudinal research that used a variety of QoL measures (subjective, objective and HRQoL) with similar populations in Europe, including the UK, as well as Canada [e.g., (31–33)]. This reinforces the role of quality of life as an important service outcome associated with recovery for this population [e.g., (34)].

The highest proportions of inadequately met needs were counseling and social needs, as well as needs related to work or using one's time, as reported in similar populations elsewhere (16, 35). This finding is also consistent with a qualitative study using the same instrument, in which Australians receiving general practice and community mental health services commonly reported their mental-health related needs for talking therapies and assistance with work/time use and social contacts were often unmet (22). Unmet needs also tend to reflect domains in which there are issues with availability or access to services (35). Considering the Australian Government's Productivity Commission (36) statement that “housing, employment services and services that help a person engage with and integrate back into the community, can be as, or more, important than healthcare in supporting a person's recovery [p2],” the accessibility of services directly addressing these unmet needs requires greater attention.

Unmet need in the domain of social relationships is associated with poor quality of life in persons with severe mental disorders, including schizophrenia (31, 32). Further, quality of life in this same population is strongly predicted by perceptions of loneliness (37). We found that feelings of loneliness and/or social isolation were strongly associated with unmet needs across all domains, except for financial needs. Loneliness and isolation are increasingly recognized as significant public health issues for the general population (38) and for this specific population group (39, 40). These factors are also rated amongst their most significant challenges by people living with psychoses themselves (41). Relationships between loneliness, poor health and increased service use are commonly emphasized [e.g., 42]. Our finding that loneliness is associated with diverse unmet psychosocial needs is therefore noteworthy since mental health interventions to directly address loneliness and isolation are few (40, 43). Arguably, isolation was related to most needs being inadequately met because we are social beings, and we know that higher reciprocity/social capital (opposite to isolation) enhances multiple aspects of our biopsychosocial lives (44, 45). It is difficult to see how individuals can address their needs when socially isolated. Recent promising developments include evidence for interventions to support increased social connectedness and community participation such as social prescribing, Clubhouses and Recovery Colleges as well as the potential for peer-delivered and peer-led interventions to address loneliness and isolation (46–49). In addition, although infrequently implemented in practice, family psychoeducation programs are well-established and known to strengthen relationships for people living with psychotic disorders within and outside the family (50). Taken together, our findings suggest that greater attention to identifying and responding to needs for social connectedness in this population holds potential for increasing wellbeing as well as supporting recovery (34, 42, 51).

Disability is reflective of challenges that people experience in everyday life in the context of complex health conditions and accompanying social disadvantages. Hence, people living with psychotic disorder and disability might be expected to identify needs for support in areas such as self-care, social connections, and activity engagement and may live in formally managed accommodation, if not living with family. The findings from this population-based study indicate that self-care needs were more likely to be met in formally managed accommodation or when someone provided this support, whereas disability overall, as measured by MSIF, was associated with unmet social, work or time use and housing-related needs. This indicates that the provision of support in these areas is not only inadequate, as noted elsewhere (16, 35) but also less well targeted toward people with psychosocial disability than self-care support (52). This has yet to be addressed within the person-centered planning and individualized funding processes of Australia's National Disability Insurance Scheme (53, 54), but also needs a coordinated approach with sectors such as health, housing and employment to tackle social exclusion among people living with psychosocial disability (55).

The unmet need in the PNCQ domain of work/time use represents a lack of meaningful ways to use time through work or activities beyond home that facilitate connection with others (22). These were amongst the highest proportion of inadequately met needs, not only suggesting the desire for meaningful use of time, but also underscoring previous time use studies that report limited participation in vocation-related, social and leisure occupations internationally (56–58). In our study, younger people were more likely to report unmet work and time use related need, as well as financial need. These needs might at least be partially met through more widespread implementation of effective vocational supports than is presently the case (59, 60). Evidence suggests that social and vocational function can continue to improve past the remittance of clinical symptoms (61). Better access is also required to individualized support for community participation that enables social connectedness, wellbeing, and recovery (47, 62, 63). Furthermore, given survey participants were typically in contact with public specialized mental health services, our findings suggest a more strongly person-centered and recovery-oriented approach is required within these services.

The strengths of this study include the large population-based sample and the use of the PNCQ, a reliable and valid needs assessment instrument. The comprehensive SHIP interview covered many aspects of participants' experiences which enabled examination of associations of personal, clinical, and psychosocial factors with perceived need in this one study. Nonetheless there were limitations. The cross-sectional nature of the SHIP survey means that the directionality of the identified associations cannot be determined and therefore, causality cannot be deduced. A relatively small proportion of the variance was explained by each model – nonetheless the positive predictive value of the models, averaging 73% was good. While it was established that multiple needs were common, survey participant views on priorities among their needs were not examined and would be a useful focus of future research. Finally, survey participants were recruited from public mental health services and so the findings may be less applicable to people receiving private mental health services in Australia.

## Conclusions

Conclusions to be drawn are that many consumers view their needs for care as unmet across many life areas despite being in contact with mental health services. Loneliness, unmet psychosocial needs, and quality of life appear strongly interconnected and warrant greater attention in the delivery of person-centered care, especially for people living with psychosis. Support to address social, work or time use and housing related needs among people living with psychosis appears less well targeted toward those with disability for which both a more coordinated intersectoral approach and greater attention by Australia's National Disability Insurance Scheme will be required. The needs of younger people living with psychosis, particularly those related to work, time use and finances, might be usefully addressed by more accessible and widely available vocational services. Together, our results further underscore the link between quality of life, recovery and needs, and these inter-relationships should be considered for all mental health services research and evaluation.

## Data availability statement

The datasets presented in this article are not readily available because access to the data and materials on which this study is based is authorized through the SHIP Access and Publication committee. The Committee Convenor is Professor Vera Morgan (vera.morgan@uwa.edu.au) and Executive Officer is Assistant Professor Anna Waterreus (anna.waterreus@uwa.edu.au). Requests to access the datasets should be directed to vera.morgan@uwa.edu.au and anna.waterreus@uwa.edu.au.

## Ethics statement

The original study was approved by all (multiple/numerous) university and health service network human Ethics Committees. The patients/participants provided their written informed consent to participate in this study.

## Author contributions

All authors contributed to the conception and design of the study. CM organized the database, performed the statistical analyses, and wrote the first draft of the manuscript. CH and EF wrote sections of the manuscript. All authors contributed to the conception and design of the study, manuscript revision, read, and approved the submitted version.

## Funding

The authors declare that the original study was funded by the Australian Government Department of Health and Aging. The funder was not involved in the study design, collection, analysis, interpretation of data, the writing of this article or the decision to submit it for publication.

## Conflict of interest

The authors declare that the research was conducted in the absence of any commercial or financial relationships that could be construed as a potential conflict of interest.

## Publisher's note

All claims expressed in this article are solely those of the authors and do not necessarily represent those of their affiliated organizations, or those of the publisher, the editors and the reviewers. Any product that may be evaluated in this article, or claim that may be made by its manufacturer, is not guaranteed or endorsed by the publisher.
